# Potential Value of Radiomics in the Identification of Stage T3 and T4a Esophagogastric Junction Adenocarcinoma Based on Contrast-Enhanced CT Images

**DOI:** 10.3389/fonc.2021.627947

**Published:** 2021-03-03

**Authors:** Xu Chang, Xing Guo, Xiaole Li, Xiaowei Han, Xiaoxiao Li, Xiaoyan Liu, Jialiang Ren

**Affiliations:** ^1^ Department of Radiology, Heping Hospital Affiliated to Changzhi Medical College, Changzhi, China; ^2^ Department of Radiology, Graduate School of Changzhi Medical College, Changzhi, China; ^3^ Department of Radiology, The Affiliated Drum Tower Hospital of Nanjing University Medical School, Nanjing, China; ^4^ Department of Pharmaceutical Diagnostics, GE Healthcare China, Beijing, China

**Keywords:** esophagogastric junction adenocarcinoma, American Joint Committee on Cancer, gastric cancer, Tumor-Node-Metastasis 8th edition, Union for International Cancer Control classification, radiomics

## Abstract

**Purpose:**

This study was designed to evaluate the predictive performance of contrast-enhanced CT-based radiomic features for the personalized, differential diagnosis of esophagogastric junction (EGJ) adenocarcinoma at stages T3 and T4a.

**Methods:**

Two hundred patients with T3 (n = 44) and T4a (n = 156) EGJ adenocarcinoma lesions were enrolled in this study. Traditional computed tomography (CT) features were obtained from contrast-enhanced CT images, and the traditional model was constructed using a multivariate logistic regression analysis. A radiomic model was established based on radiomic features from venous CT images, and the radiomic score (Radscore) of each patient was calculated. A combined nomogram diagnostic model was constructed based on Radscores and traditional features. The diagnostic performances of these three models (traditional model, radiomic model, and nomogram) were assessed with receiver operating characteristics curves. Sensitivity, specificity, accuracy, positive predictive value, negative predictive value, and areas under the curve (AUC) of models were calculated, and the performances of the models were evaluated and compared. Finally, the clinical effectiveness of the three models was evaluated by conducting a decision curve analysis (DCA).

**Results:**

An eleven-feature combined radiomic signature and two traditional CT features were constructed as the radiomic and traditional feature models, respectively. The Radscore was significantly different between patients with stage T3 and T4a EGJ adenocarcinoma. The combined nomogram performed the best and has potential clinical usefulness.

**Conclusions:**

The developed combined nomogram might be useful in differentiating T3 and T4a stages of EGJ adenocarcinoma and may facilitate the decision-making process for the treatment of T3 and T4a EGJ adenocarcinoma.

## Introduction

In 1996, Siewert et al. defined esophagogastric junction (EGJ) adenocarcinoma as tumors with a center located within 5 cm proximal and distal to the anatomical cardia (1). In recent decades, the incidence of EGJ adenocarcinoma has shown a significant increasing trend (2). A study conducted in China also showed that the incidence of EGJ adenocarcinoma increased from 22.3 to 35.7% from 1988 to 2012 (3). A histological transition is observed at the junction of the esophagus and stomach, and its pathological changes and biological behavior are different from the esophagus and stomach (4). EGJ is therefore defined, staged, and treated as a unique zone. According to the American Joint Committee on Cancer (AJCC) Cancer Staging Manual 8th edition, the anatomical boundary of EGJ tumors was defined as follows: “tumors involving the EGJ with the tumor epicenter no more than 2 cm into the proximal stomach are staged as esophageal cancers; EGJ tumors with their epicenter located greater than 2 cm into the proximal stomach are staged as stomach cancers” (5). According to the manual, Siewert type III EGJ adenocarcinoma uses gastric cancer staging, while type I and type II EGJ adenocarcinoma still uses esophageal cancer staging (6).

Accurate preoperative clinical staging plays an important role in determining the treatment strategy for patients. The American Joint Committee on Cancer/Union for International Cancer Control (AJCC/UICC) recommended computed tomography (CT) of the chest and abdomen with oral and intravenous contrast as an important modality for the clinical Tumor-Node-Metastasis (TNM) staging of advanced upper gastrointestinal tumors. However, it also suggests that CT plays a limited role in determining the primary tumor category (cT), while the identification of cT3 and cT4 is the major limitation (5). The accuracy of preoperative CT in the discrimination of T3 and T4a disease in patients with Siewert II EGJ adenocarcinoma was 74.4% (32/43) using the UICC/AJCC criteria (7). The total accuracy of multislice spiral CT (MSCT) for determining the T stage of Siewert type II and III EGJ adenocarcinoma was 63.5% (8). The degree of tumor invasion is an important prognostic factor for EGJ adenocarcinoma, particularly locally advanced cancer classified as T4a according to the TNM classification. For T4a EGJ adenocarcinoma, neoadjuvant chemotherapy is beneficial to reduce the tumor grade before resection and micrometastasis treatment (9). Therefore, the accurate differentiation of T3 and T4a will aid in the selection of a better treatment strategy.

An urgent need is to find new methods to improve the differentiation between T3 and T4a EGJ adenocarcinoma. The application of artificial intelligence radiomics as a bridge between medical imaging and individualized medicine has the potential to solve existing problems associated with a subjective imaging diagnosis (10, 11). Radiomics transforms imaging data into high-dimensional minable feature sets through a series of data characterization algorithms to explore tumor heterogeneity and the microenvironment (12, 13). This approach has been shown to be useful in evaluating and predicting the histopathological features, treatment response, and prognosis of tumors (12, 14).

In this study, we hypothesized that the CT radiomic features of preoperative EGJ adenocarcinoma may provide valuable information for the differentiation of T3 and T4a disease. We performed a radiomic analysis of CT images from 200 patients with surgically confirmed T3 and T4a EGJ adenocarcinoma. The objective of this study was to establish a reliable radiomic model for differentiating T3 and T4a EGJ adenocarcinoma.

## Materials and Methods

### Study Design

**Figure 1:**
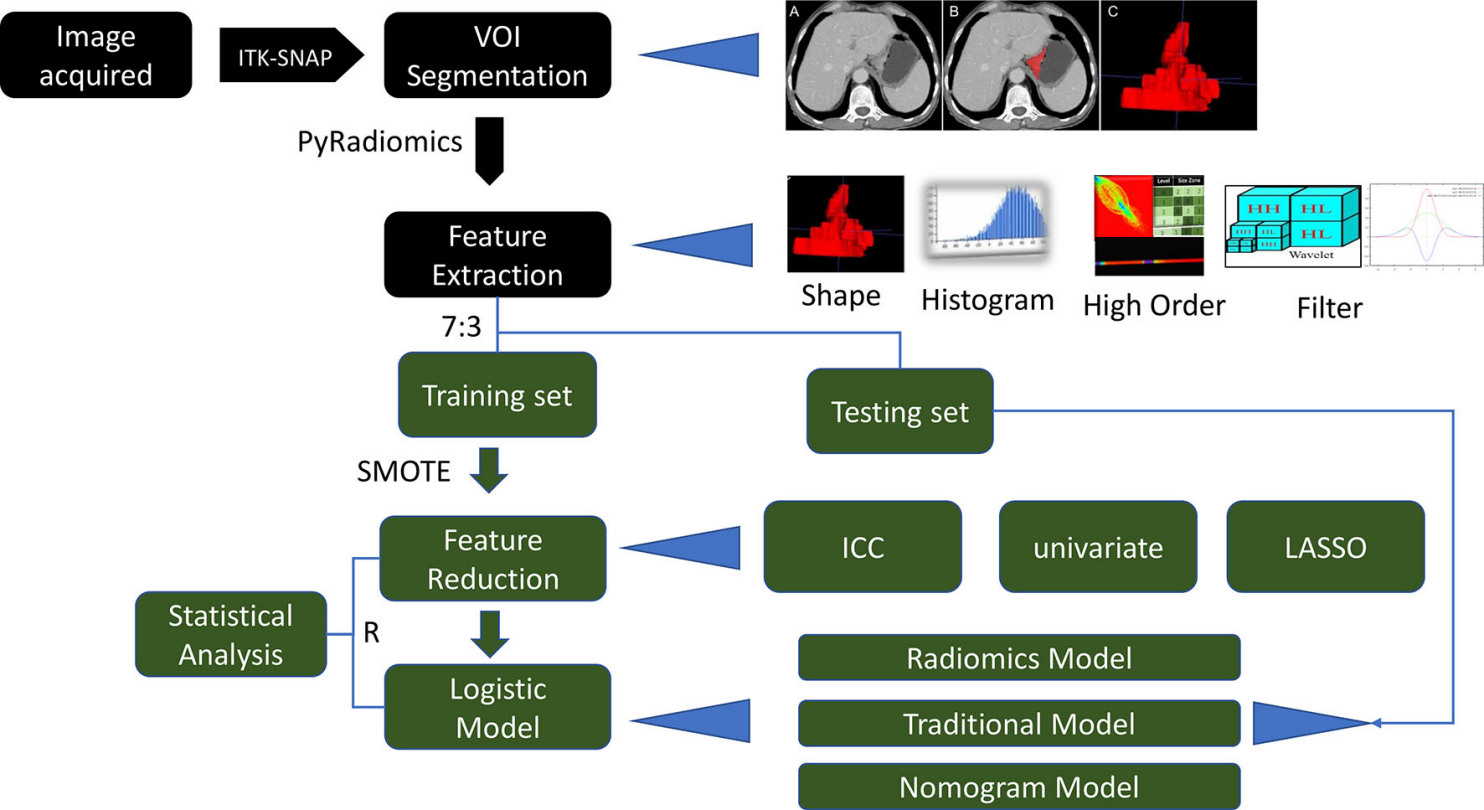
Radiomic model and workflow of the analysis performed in this study.

The main objective of this study was to evaluate the predictive performance of contrast-enhanced CT-based radiomic features for the personalized, differential diagnosis of T3 and T4a EGJ adenocarcinoma. This study further explored whether the combination of radiomic and traditional features would further improve the accuracy of model performance. Radiomic features were obtained from the segmentation area of CT images. The data were divided into training and test sets for model training and independent validation, respectively. A series of feature selection methods were used to mine the most valuable radiomic features. Finally, a logistic regression method was used to build a predictive model. This study included five parts, including CT image acquisition, image analysis, volume of interest (VOI) segmentation, feature extraction, and model building. A detailed description of the radiomic model and analysis workflow is shown in **Figure 1**.

### Patients

The Institutional Review Board of Heping Hospital Affiliated to Changzhi Medical College approved this retrospective study and waived the requirement to obtain informed consent. All methods were performed in accordance with the guidelines and regulations of this ethics board. The inclusion criteria were as follows: (a) EGJ adenocarcinoma confirmed by postoperative pathology (according to AJCC Cancer Staging Manual 8th edition diagnostic criteria of EGJ cancer); (b) an electronic gastroscopy examination and contrast-enhanced abdominal CT examination performed within 2 weeks before surgery; and (c) radical resection of the tumor within 2 weeks after the contrast-enhanced CT examination. The exclusion criteria were as follows: (a) neither stage T3 nor T4a EGJ adenocarcinoma was determined by postoperative pathology; (b) any local or systematic treatment before surgery, such as neoadjuvant chemotherapy; (c) no complete thin-slice images; (d) poor image quality, such as poor visualization of the peristaltic motion, insufficient distention of the stomach, or hiatal hernia; and (e) the edge of the tumor was difficult to define.

### CT Image Acquisition

The following specifications of the stomach protocol were used in this study: the patients were asked to fast for 8 to 12 h before the examination; the patients were also encouraged to drink 750–1,000 ml of warm water 10 min prior to the CT scan and an additional 250 ml of warm water prior to image acquisition. A Siemens dual-source CT system (SOMATOM Definition) was used in this study. The scanning range was from the superior diaphragmatic 5 cm (inferior pulmonary vein) to the lower margin of both kidneys or the superior margin of the pubic symphysis. After the unenhanced scan was completed, 1.5 ml/kg of an iodine contrast agent (Omnipaque, GE Healthcare, 100 ml:35 g) was injected through the anterior elbow vein at a flow rate of 3.0 ml/s using a high-pressure syringe (German Ulrich Missouri double-barrel high-pressure syringe). The automatic trigger mode was used to scan the arterial phase, and the trigger threshold was 100 HU. After the completion of the arterial-phase scanning, dynamic enhanced scanning was performed in the venous phase and delayed phase for 25 s and 180 s, respectively. The following scanning parameters were used: tube voltage, 120 kVp; tube current, 220 mA; automatic millisecond technology, on; pitch, 0.8; collimator layer thickness, 0.6 mm; and rotation time, 0.33 s.

### Image Analysis

**Figure 2:**
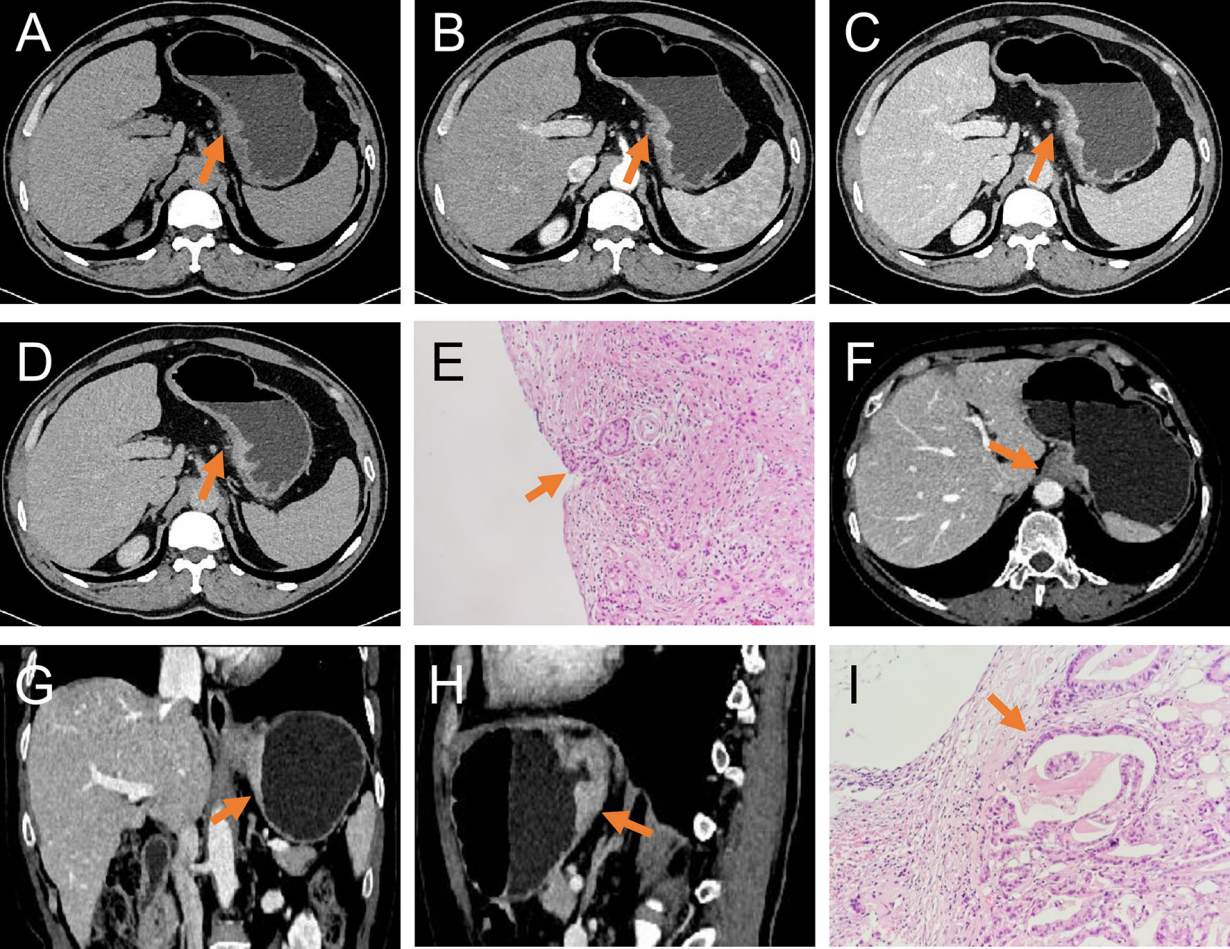
T3 and T4a EGJ adenocarcinoma. **(A–D)** Axial unenhanced, arterial-phase, venous-phase, and delayed-phase CT images, respectively, of T4a EGJ adenocarcinoma with a nodular shape of the serous surface and infiltration of surrounding fat (arrows). **(E)** The histopathological analysis under light microscope showed that the tumor invaded the serosa (arrows). **(F–H)** Axial, coronal, and sagittal venous-phase CT images of T3 EGJ adenocarcinoma with a smooth serous surface (arrows). **(I)** The histopathological analysis under the light microscope showed that the tumor invaded the subserosal connective tissue (arrows).

The data were transferred to a syngo.via for Oncology, Siemens workstation (VB20) after the scan was completed, and the contrast-enhanced images were reconstructed with 1.00-mm-thick cross-sections. Two radiologists (CX and GX with 4 years and 20 years of experience, respectively, in clinical abdominal CT diagnosis) with expertise in picture archiving and communication system (PACS) retrieval blindly and independently read the T3 and T4a staging signs without knowing the pathological results. According to the AJCC Cancer Staging Manual 8th edition diagnostic criteria of EGJ cancer, tumors involving the EGJ with the tumor epicenter extending no more than 2 cm into the proximal stomach were staged as esophageal cancers and tumors with the epicenter located greater than 2 cm into the proximal stomach were staged as stomach cancer (5). T3 and T4a staging was performed according to the Chinese Society of Clinical Oncology (CSCO) gastric cancer guidelines 2019 (15): T3—conventional reference signs (highly enhanced cancer invasion in the whole layer of the gastric wall, smooth serosa surface, or a few short cords), auxiliary reference signs (blurred serosa or short stripes <1/3 the total lesion area) (16, 17); T4a—conventional reference signs (irregular or nodular shape of the serous surface, dense burr, or banded infiltration of the surrounding fat space), auxiliary reference signs (serous high enhancement line sign, fault zoning method) (18, 19), as shown in **Figure 2**. The traditional CT signs were evaluated as follows (1): smooth serous surface; (2) a few short cords of the serous surface; (3) the fat space around the tumor was clear; (4) blurred serosa or short stripes <1/3 the total lesion area; (5) irregular or nodular shape of the serous surface; (6) dense burr or banded infiltration of the surrounding fat space; (7) disappearance of the fat space between the serosa and peripheral vessels; (8) cyst degeneration or necrosis: low-density cystic degeneration or necrosis in the tumor tissue; (9) tumor thickness: measurement of the maximum thickness of the tumor, namely, the maximum vertical distance from the surface of the lesion to the deepest infiltration; and (10) longest diameter: the longest diameter in the multiplanar reconstruction (MPR) was measured from the top of the highest lesion to the bottom of the lowest lesion.

In the event that the two diagnostic radiologists recorded different subjective EGJ adenocarcinoma characteristics, they negotiated a repeated evaluation and reached a consensus. The average thickness and longest diameter of the tumor measured by the two doctors were used.

### Tumor Segmentation

**Figure 3:**
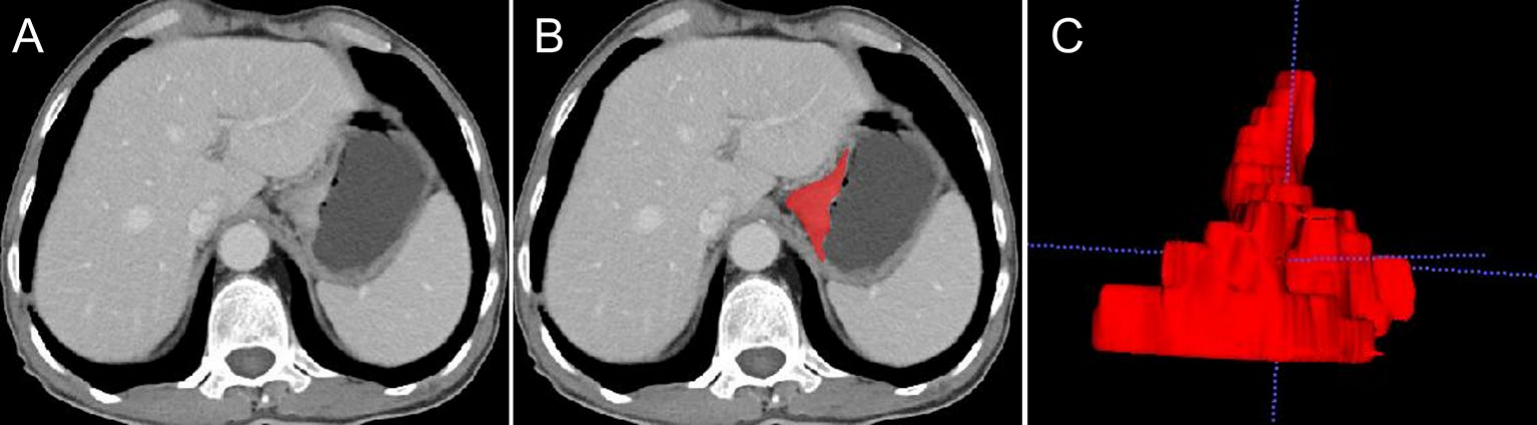
An example of manual segmentation of EGJ adenocarcinoma. **(A)** A localized mass with heterogeneous enhancement is observed at the EGJ on the venous-phase CT image. **(B)** Segmentation of the same axial slice. **(C)** 3D volumetric reconstructions of the tumor generated from ITK-SNAP.

The tumor lesions of the EGJ were segmented manually on venous-phase images with a slice thickness of 5 mm using ITK-SNAP software (www.itksnap.org, version: 3.8.0). The gastric cancer (GC) lesions were manually identified by a radiologist (CX with 4 years of abdominal imaging diagnosis experience) and confirmed by another abdominal radiologist (GX with 20 years of abdominal imaging diagnosis experience). Neither radiologist had access to the clinicopathological characteristics of the patient. GC was defined as focal thickening of the gastric wall with a thickness of at least 6 mm and significant enhancement (20). The region of interest (ROI) profile was segmented along the boundary of the tumor in each slice and a fixed soft tissue window (window width, 400 HU; window level, 40 HU) was set to avoid artifacts, perigastric vessels, and the gastric cavity. Thirty cases were randomly selected and segmented again for use in the analysis of intragroup correlation consistency. The whole tumor segmentation process was completed layer by layer, and the 3-dimensional (3D) model of the lesion and VOI was obtained. **Figure 3** shows an example of the manual segmentation of EGJ adenocarcinoma. Finally, each VOI was examined by GX.

### Radiomic Feature Extraction

The data were divided into a training set and test set at a ratio of 7:3 with a stratified sampling strategy. PyRadiomics (open source imaging toolbox) was used to extract the features of the segmented images. In addition, two image filters of Laplacian of Gaussian (LoG, sigma value: 3, 5) and wavelet were applied to the original image, and the corresponding derived images were generated. All categories of radiomic features, except shape features, were extracted from the original images and 10 filtered images. Eighteen first-order histogram features, 14 shape (3D morphological) features, 24 gray co-occurrence matrix (GLCM) features, 16 gray-level run length matrix (GLRLM) features, 16 gray-level size zone matrix (GLSZM) features, 5 neighboring gray tone difference matrix (NGTDM) features, and 14 gray-level dependence matrix (GLDM) features were obtained. Because of the deviation in the sample size between patients with T3 and T4a tumors, which may influence the performance of classifier, the synthetic minority oversampling technique (SMOTE) algorithm with default parameters was used to improve the imbalance in the data, where minority instances were generated along a line joining a minority instance and its nearest neighbors (21). Original data and SMOTE amplification data distributions are shown in **Supplemental Table 1**.

The following radiomic feature preprocessing steps were performed: (1) the missing value was replaced with the median value; and (2) each feature was standardized by the z-score method. The following feature selection method was performed to reduce the redundancy of features: (1) retain the features with good intragroup correlation coefficient (ICC) consistency; (2) remove the features with a correlation greater than 0.7; (3) remove the features with p > 0.05 in the univariate logistic regression analysis; and (4) select the important features using the least absolute shrinkage and selection operator (LASSO) method.

### Model Building

A univariate analysis was used to evaluate the traditional CT signs related to the differential diagnosis of T3 and T4a EGJ adenocarcinoma. A multivariate logistic regression analysis was used to build traditional model and radiomic model. A combined nomogram that integrated both the radiomic score (Radscore) and the traditional features in the training set was further built. The Radscore was calculated based on the radiomic model in the training set, and a box plot was used to show the distribution of the Radscores. The test set was used to validate those models.

### Statistical Analysis

The statistical analysis was performed using R (www.r-project.org, version: 3.6.1). The SMOTE method was performed with the “DMwR” R packages. The ICC was calculated to evaluate the consistency of the radiomic features after different VOI segmentations. An ICC >0.75 indicated good consistency. The diagnostic performance of the three models was evaluated by constructing the receiver operating characteristic curve (ROC). Optimal cutoff points were obtained by calculating the Youden index. Then, the area under the ROC curve (AUC), accuracy, sensitivity, specificity, positive predictive value, and negative predictive value were calculated (22). The Wilcoxon signed rank test was used to compare the discrimination efficiency of the Radscore for T3 and T4a EGJ adenocarcinoma. A decision curve analysis (DCA) was employed to evaluate the clinical utility of the three models. The DeLong test was used for statistical comparisons of the ROC curves (23, 24). ROC curves and Delong tests were generated and performed with the “pROC” R package. Two-tailed p-values less than 0.05 were considered statistically significant.

### Clinical Characteristics of the Patients

Two hundred patients were included in this study, including 44 and 156 patients with T3 and T4a EGJ adenocarcinoma confirmed by surgical pathology from December 2017 to March 2019, respectively. The demographic data and T stages of the patients with T3 and T4a EGJ adenocarcinoma are listed in **Table 1**. The median (range) ages of the two sets were 64 (49–87) years and 63 (43–82) years, respectively, and the proportion of males was 16.5 and 63.5%, respectively. The coincidence rate of clinical T stage and pathological T stage in T3 and T4a EGJ adenocarcinoma was 9.5 and 66%, respectively. Notably, 12% of patients with T3 tumors were overestimated as having T4a tumors, and 12.5% of patients with T4a tumors were underestimated as having T3 tumors based on the clinical stage.

**Table 1:** Features obtained after preprocessing.

Variables	T3	T4a
Sex		
Male	33 (16.5%)	127 (63.5%)
Female	11 (5.5%)	29 (14.5%)
Age (Y)	64 (49-87)	63 (43-82)
T stage		
Pathological T stage	44 (22%)	156 (78%)
Clinical T stage	43 (21.5%)	157 (78.5%)
Coincidence	19 (9.5%)	132 (66%)
Overestimation Underestimation	24 (12%)	25 (12.5%)

### Traditional and Radiomic Features

**Figure 4:**
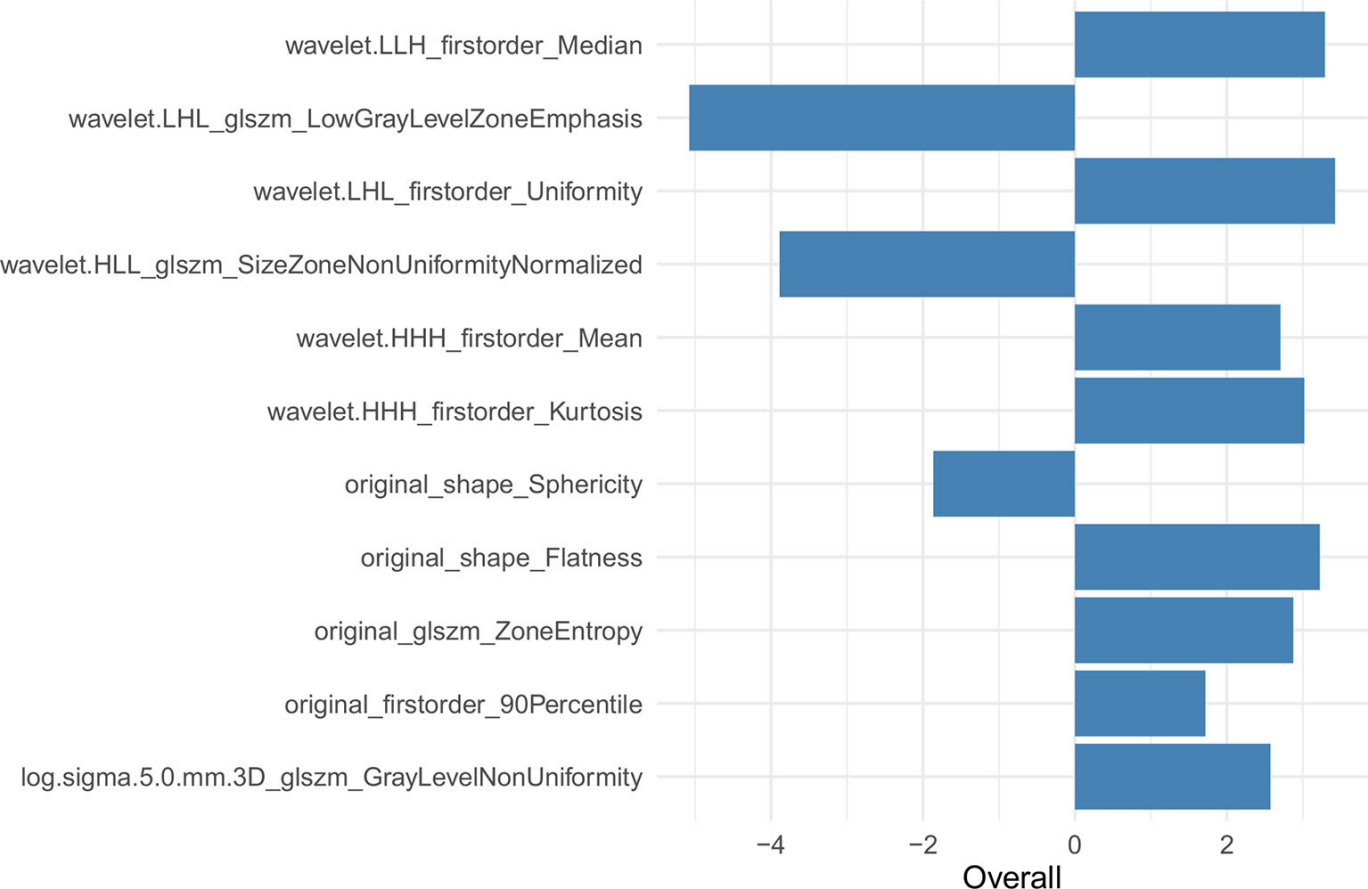
Weights of 11 retained radiomic features in the radiomic data.

The univariate analysis showed that a few short cords on the serous surface and a clear fat space around the tumor were independent predictors of T3 and T4a among the 10 traditional CT signs (**Table 2**). The statistics of traditional CT signs between the training and test groups are shown in **Supplemental Table 2**. Overall, 1,037 radiomic features were extracted from each VOI, and 921 features with an ICC >0.75 were retained by the consistency analysis. Eleven important radiomic features were retained after dimension reduction (**Supplemental Table 3**). The 11 retained important radiomic features included five first-order features (median, uniformity, mean, kurtosis, and 90th percentile), two shape features (flatness and sphericity), and four GLSZM features (low gray-level zone emphasis, LGLZE; size-zone non-uniformity normalized, SZNN; zone entropy, ZE; and gray-level non-uniformity, GLN). **Supplemental Table 4** shows the radiomic features in the original data and SMOTE radiomic data and beta values (regression coefficient) of the features. **Supplemental Table 5** lists the equations of the 11 features, and **Figure 4** shows the weights of the retained radiomic features.

**Table 2:** Univariate logistic regression analysis of traditional features between T3 and T4a EGJ adenocarcinoma in the training and test sets.

Features	Training data			Test data		
	T3	T4a	p	T3	T4a	p
n	31	110		13	46	
Age, Y [mean (SD)]	63.58 (6.82)	63.25 (8.35)	0.842	65.08 (8.16)	63.65 (7.72)	0.564
Sex = 1 (%)	23 (74.2)	89 (80.9)	0.572	10 (76.9)	38 (82.6)	0.951
Serosa Smooth = 1 (%)	8 (25.8)	11 (10.0)	0.048	4 (30.8)	2 (4.3)	0.024
Serosa Cords = 1 (%)	6 (19.4)	6 (5.5)	0.037	1 (7.7)	4 (8.7)	1.000
Fat Clear = 1 (%)	7 (22.6)	10 (9.1)	0.085	4 (30.8)	2 (4.3)	0.024
Serous Blurred = 1 (%)	7 (22.6)	7 (6.4)	0.020	1 (7.7)	4 (8.7)	1.000
Serosa Nodular = 1 (%)	17 (54.8)	93 (84.5)	0.001	8 (61.5)	40 (87.0)	0.094
Fat Blurred = 1 (%)	17 (54.8)	93 (84.5)	0.001	8 (61.5)	40 (87.0)	0.094
Fat Disappearance = 1 (%)	12 (38.7)	61 (55.5)	0.149	4 (30.8)	28 (60.9)	0.108
Necrosis = 1 (%)	12 (38.7)	50 (45.5)	0.643	6 (46.2)	21 (45.7)	1.000
Thickness [median (IQR)]	17.92 [13.55, 20.48]	18.09 [14.57, 23.95]	0.133	17.93 [12.82, 19.20]	18.95 [14.18, 21.92]	0.297
Longest Diameter [median (IQR)]	45.92 [36.29, 62.23]	59.72 [49.63, 77.03]	0.001	52.44 [37.65, 68.87]	59.73 [49.47, 71.02]	0.380

Serosa Smooth: smooth serous surface; Serosa Cords: a few short cords on the serous surface; Fat Clear: the fat space around the tumor was clear; Serous Blurred: blurred serosa or short stripes <1/3 the total lesion area; Serosa Nodular: irregular or nodular shape of the serous surface; Fat Blurred: dense burr or banded infiltration of the surrounding fat space; Fat Disappearance: disappearance of the fat space between the serosa and peripheral vessels; Necrosis: cyst degeneration or necrosis; Thickness: tumor thickness; Longest Diameter: the longest diameter of the tumor.

### Combined Nomogram Construction

**Figure 5:**
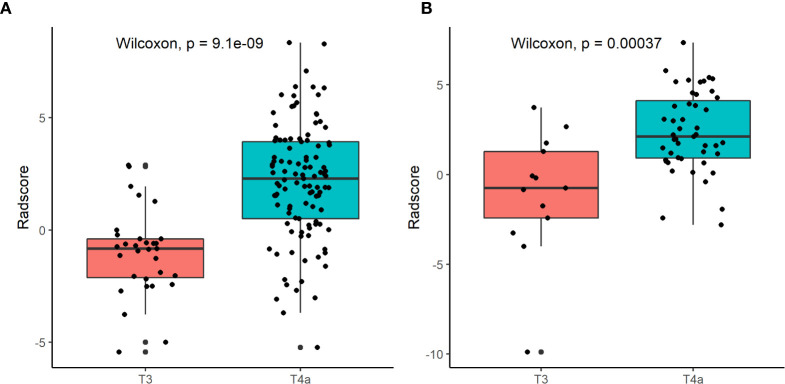
Box plots of Radscore distributions in the training set **(A)** and the test set **(B)** using the radiomic model.

**Figure 6:**
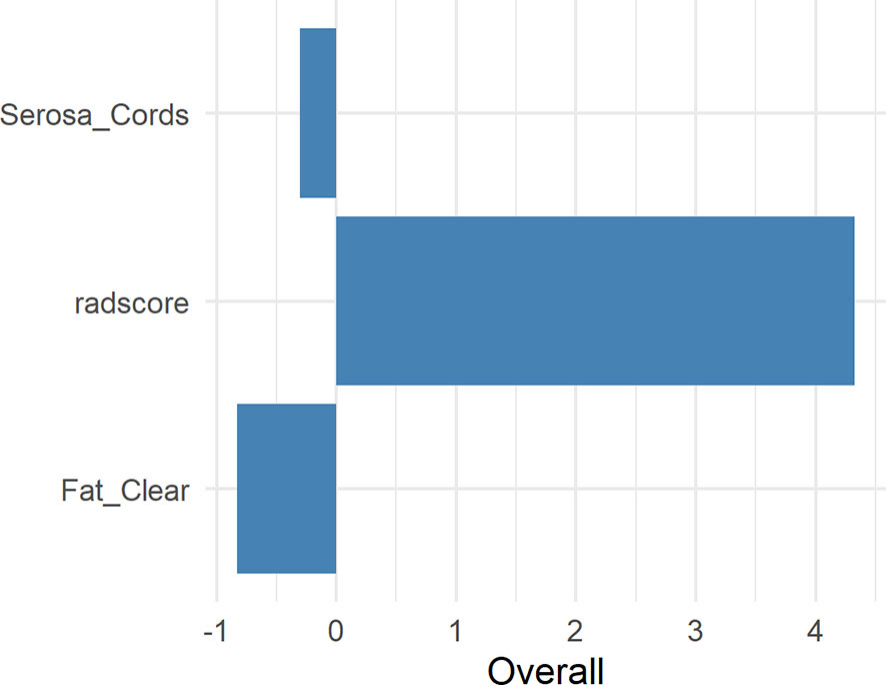
Weights of the Radscore, Serosa Cords, and Fat Clear. Serosa Cords: a few short cords on the serous surface; Fat Clear: the fat space around the tumor was clear.

**Figure 7:**
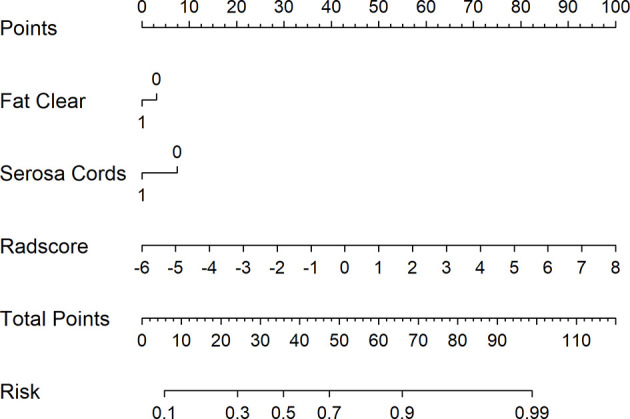
Combined nomogram to identify T3 and T4a EGJ adenocarcinoma. For instance, for a patient with Fat Clear, Serosa Cords, and Radscore of −2, the total points were 0 + 0 + 30 = 30. The score of 30 is decreasing compared with the risk of 0.4, indicating that the T stage of this patient is more likely to be T3. Serosa Cords: a few short cords on the serous surface; Fat Clear: the fat space around the tumor was clear.

**Figure 5** shows the difference in the Radscore distribution between the training and test sets, with a low value for T3 and a high value for T4a. The statistical results obtained from the test set showed a significant difference in the Radscore between T3 and T4a (p = 0.00037). **Figure 6** shows the weights of the features included in the combined nomogram, and the feature weight of the Radscore was significantly higher than that of serosa cords and fat. We further visualized the results of the multivariate analysis of the identification of stage T3 and T4a EGJ adenocarcinoma using a combined nomogram model, as shown in **Figure 7**. The variables in the combined nomogram model included the Radscore, a few short cords on the serous surface and a clear fat space around the tumor. The risk indicated in the nomogram represents the probability of a T4a tumor.

### ROC Curve Analysis and DCA

A ROC curve analysis was conducted to evaluate the sensitivity and specificity of the combined nomogram model {AUC: 0.845 [95% confidence interval (CI), 0.772–0.918] and 0.812 [95% CI, 0.669–0.954]; sensitivity: 0.835 and 0.936; specificity: 0.839 and 0.692}, the radiomic model [AUC: 0.839 (95% CI, 0.767–0.911) and 0.812 (95% CI, 0.670–0.953); sensitivity: 0.817 and 0.915; specificity: 0.839 and 0.538], and the traditional feature model [AUC: 0.645 (95% CI, 0.550–0.741) and 0.613 (95% CI, 0.467–0.759); sensitivity: 0.862 and 0.851; specificity: 0.419 and 0.385] in the training and test sets and to substantiate the value of radiomics in the differential diagnosis of stage T3 and T4a EGJ adenocarcinoma. The combined nomogram model exhibited the highest accuracy, as shown in **Table 3**. **Supplemental Figure 1** shows the ROC curves for the four models. The data were more balanced with the SMOTE algorithm, and the efficiency of the SMOTE radiomic model was better than the original model.

**Table 3:** Performance of the four models.

Model	Training set						Test set					
	AUC	ACC	Sen	Spe	PPV	NPV	AUC	ACC	Sen	Spe	PPV	NPV
Radiomic	0.758 (0.650–0.866)	0.779 (0.701–0.844)	0.817	0.645	0.890	0.500	0.781 (0.597–0.964)	0.683 (0.55–0.797)	0.681	0.692	0.889	0.375
SMOTE Radiomic	0.839 (0.767–0.911)	0.821 (0.748–0.881)	0.817	0.839	0.947	0.565	0.812 (0.67–0.953)	0.833 (0.715–0.917)	0.915	0.538	0.878	0.636
Traditional	0.645 (0.550–0.741)	0.764 (0.685–0.832)	0.862	0.419	0.839	0.464	0.613 (0.467–0.759)	0.750 (0.621–0.853)	0.851	0.385	0.833	0.417
Nomogram	0.845 (0.772–0.918)	0.836 (0.764–0.893)	0.835	0.839	0.948	0.591	0.812 (0.669–0.954)	0.883 (0.774–0.952)	0.936	0.692	0.917	0.750

AUC, area under the ROC curve; ACC, accuracy; Sen, sensitivity; Spe, specificity; PPV, positive predictive value; NPV, negative predictive value.

**Figure 8:**
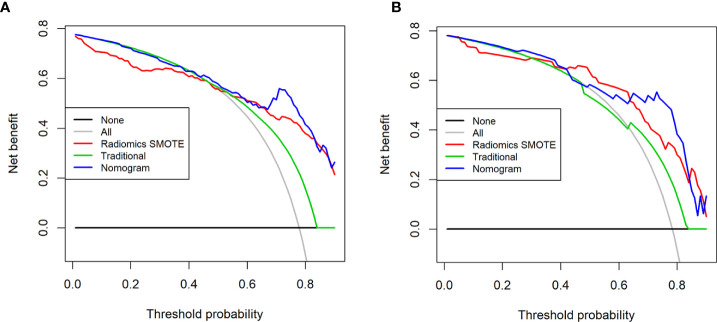
Decision curves for the three models in the training set **(A)** and the test set **(B)**. The x-axis of the decision curve is the threshold of the predicted probability obtained using the three models (traditional model, SMOTE radiomic model, combined nomogram) to identify T3 and T4a EGJ adenocarcinoma, and the y-axis reflects the clinical decision of a net benefit for patients based on the classification result at this threshold. The decision curves for the “all discrimination” scheme and the “no discrimination” scheme were used as references in the DCA. The area under the decision curve shows the clinical practicability of the three models.

Decision curves for the three models are presented in **Figure 8**. The DCA showed that when the threshold was greater than 0.7 in the training set, the net benefit of clinical decision-making provided by the combined nomogram model was higher than the other models. The combined nomogram model also produced the largest AUC and best clinical practicability. All the results were verified in the test set.

## Discussion

In this study, we extracted and validated the feasibility of a preoperative non-invasive differential diagnosis of T3 and T4a EGJ adenocarcinoma based on the Radscore obtained from the radiomic model. The Radscore had good predictive performance. Its capacity to distinguish T3 and T4a EGJ adenocarcinoma was significantly better than the other traditional features. The Radscore is often used to evaluate the prognosis of tumors in a texture analysis but has rarely been used for differential diagnosis in previous studies (25).

Tumors are heterogeneous at the tissue and cellular levels, as well as genetic and phenotypic levels, and are spatially heterogeneous in terms of cell density, angiogenesis and necrosis. This type of tumor heterogeneity may be related to the biologically invasive behavior (26, 27). Texture analysis provides an objective and quantitative assessment of tumor heterogeneity by analyzing the distribution and interrelationship of pixel or voxel gray levels in the image (28, 29). This method was applied to tumor samples, but the selected samples should represent tumor heterogeneity as much as possible. Based on the spatial heterogeneity of tumor growth and the evaluation of the degree of invasion, a whole-tumor analysis was used in this study. However, a recent study showed that models constructed with 2D radiomic features displayed comparable performances with models constructed with 3D features in characterizing GC (30).

In the current study, we extracted 11 radiomic features to identify T3 and T4a EGJ adenocarcinoma. First-order features describe the distribution of voxel intensities within the ROI. The median refers to the intermediate gray-level intensity within the ROI. Uniformity is a measure of the sum of the squares of each intensity value, which is a measure of the homogeneity of the image array. Greater uniformity implies greater homogeneity in the tumor. In our study, uniformity was positively correlated with the differentiation of EGJ adenocarcinoma into stages T3 and T4a. The LGLZE measures the distribution of lower gray-level size zones, with a higher value indicating a greater proportion of lower gray-level values and size zones in the image. A lower LGLZE value may indicate liquefaction and necrosis in the tumors, while a higher SZNN value may indicate less homogeneity among zone size volumes in the VOI. The LGLZE and SZNN values were negatively correlated, and these two features had the largest weight among the 11 features. The Radscore effectively distinguished between T3 and T4a EGJ adenocarcinoma. The diagnostic efficiency of the combined nomogram generated from the Radscore and traditional features was significantly better than the traditional model in both the training and test sets.

One of the highlights of the 8th edition of the AJCC/UICC staging guidelines was the first mention of the importance of MPR in the clinical T staging of GC (5). A study conducted by Chen et al. showed that the accuracy of T staging can be improved by 10 to 20% by combining axial, coronal, and sagittal images (31). In addition, tumor staging at the EGJ includes observing the infiltration of adjacent organs and the bare area of the stomach. EGJ cancer often grows along with the cardiac wall; therefore, multiplane observation is beneficial for comprehensively observing the depth of tumor invasion into the gastric wall and objectively evaluating GC invasion in the surrounding organs. In the present study, the evaluation of traditional signs was reconstructed by 1.00 mm in MPR. Currently, an authoritative guide to recommend the thickness of reconstruction is unavailable. The choice of a 1.00 mm layer thickness was based on the reconstruction habits of our department.

The AJCC proposed preoperative clinical TNM staging to guide attending physicians in making preliminary treatment decisions, but a lack of consistency in the initial clinical evaluation has been documented, including non-standardized radiological reports (5). Kim et al. reported that the accuracy of CT in distinguishing T3 and T4 GC was only 60%, similar to the diagnostic accuracy of the traditional model in the present study (ACC: training set, 0.764; test set, 0.750) (18). In a multicenter prospective study, the preoperative clinical stage and postoperative pathological diagnosis of 4,534 cases of GC were compared. The coincidence rate of T3 and T4 GC was 36.1 and 57.6%, respectively (32). Another study showed coincidence rates of T3 and T4 of 38.2 and 55.9%, respectively (33). In our study, T3 and T4a stages were diagnosed according to the 8th edition of the AJCC/UICC staging guidelines, and the consistency of T3 was 9.5%. The consistency of T4a was 66.0%, excessive staging was 12.0%, and staging was less than 12.5%. In this study, only T3 or T4a was interpreted, which may be the reason for the high consistency of T4a.

Liu et al. explored the correlation between CT texture parameters and TNM staging of GC (34). When early GC was compared to advanced GC, the maximum frequency of the arterial phase and venous phase showed good deviation (AUC: 0.810, 0.752, 0.822, all p < 0.05), and the enhanced information obtained in the venous phase was more closely related to the invasiveness of GC. A recent study showed the powerful diagnostic ability of a nomogram for the evaluation of serosa invasion in advanced GC in the training, internal and external validation sets, with AUCs of 0.90 (95% CI, 0.86–0.94), 0.87 (95% CI, 0.82–0.92), and 0.90 (95% CI, 0.85–0.96), respectively (35). Another study of primary EGJ adenocarcinoma showed that the shape compactness based on a radiomic texture analysis and pathological grade differentiation has great potential for pretreatment risk classification to guide surgical resection in patients with locally advanced diseases (36). In our study, the nomogram had its highest diagnostic ability in the training set and test set, with AUCs of 0.845 (95% CI, 0.772–0.918) and 0.812 (95% CI, 0.669–0.954), respectively. To date, few radiomic studies of GC and EGJ adenocarcinoma have been conducted. Therefore, we hope our research will provide some assistance to researchers in related fields.

In our study, good variable control was performed for all the patients in the group. The VOI was manually segmented based on tumor heterogeneity and repeatability. Segmentation was first performed by a radiologist and then confirmed by a senior radiologist, and the consistency of the results was evaluated before and after segmentation. After SMOTE amplification, we obtained the Radscore from the radiomic model, which performed well in the identification of T3 and T4a EGJ adenocarcinoma. We generated a combined nomogram model that integrated the Radscore and traditional CT features to provide a clinical method for the personalized differentiation of T3 and T4a EGJ adenocarcinoma. The performance of the nomogram was further verified in the test set.

Our study has several limitations. (1) The total number of patients was insufficient, and the number of T3 cases was relatively small. Although the data were amplified by statistical processing to avoid data offset in feature extraction, more samples are still required to further verify the findings. (2) For EGJ cancer, we were unable to completely follow the classifications recommended by the AJCC/UICC because of the difficulties in establishing the EGJ line and the tumor center. In addition, a unified standard for the clinical TNM staging of EGJ cancer has not been established according to the guidelines and determining the tumor center, EGJ line, and distance of EGJ at 2 cm on CT images. (3) This study only included venous-phase images and did not perform the same histological analysis of arterial-phase, delayed-phase, or unenhanced images. The Siemens dual-source CT automatic trigger mode was used to verify the tissue-level drug concentration. The use of contrast agents substantially improves recognition of the tumor edge (24). Digestive tract tumors display the best contrast in the venous phase. Liu et al. showed that the enhanced information obtained in the venous phase was more closely related to the invasiveness of GC (34). Therefore, the accurate depiction of the tumor in other phase images may be difficult, which in turn may affect the ROI delineation and ultimately affect the calculation of radiomic features (4). EGJ adenocarcinoma can be evaluated using ultrasound gastroscopy, contrast-enhanced CT, MRI, and PET/CT. This study only examined CT images, and other imaging methods should be added to establish the corresponding models of various imaging methods in future studies. This study collected data from only one center and failed to compare data from multiple centers. Thus, the generalization of the results should be investigated.

In conclusion, we identified and validated the Radscore as a powerful tool for differentiating T3 and T4a EGJ adenocarcinoma. We also proposed and verified a combined nomogram model integrating the Radscore and traditional CT features that can be easily used to accurately identify T3 and T4a tumors. Our results may facilitate the decision-making process for the treatment of T3 and T4a EGJ adenocarcinoma.

## Data Availability Statement

The raw data supporting the conclusions of this article will be made available by the authors, without undue reservation.

## Ethics Statement

The studies involving human participants were reviewed and approved by Heping Hospital Affiliated to Changzhi Medical College Institutional Review Board. Written informed consent for participation was not required for this study in accordance with the national legislation and the institutional requirements. Written informed consent was not obtained from the individual(s) for the publication of any potentially identifiable images or data included in this article.

## Author Contributions

XC and XG participated in the conceptualization of the study and revised some of the intellectual content in the manuscript. XC drafted the manuscript. XLL, XXL, and XYL collected CT images. JR participated in CT feature extraction, deployed the machine-learning algorithm, and was responsible for statistical analyses. XG and XH participated in the revision of the manuscript. All authors contributed to the article and approved the submitted version.

## Funding

This study was supported by the WU JIEPING Medical Foundation (320.6750.2020-08-7).

## Conflict of Interest

Author JR was employed by company GE Healthcare China.

The remaining authors declare that the research was conducted in the absence of any commercial or financial relationships that could be construed as a potential conflict of interest.
