# Supplementary material for: Potential Value of Radiomics in the Identification of Stage T3 and T4a Esophagogastric Junction Adenocarcinoma Based on Contrast-Enhanced CT Images
Source: Front Oncol. 2021 Mar 3;11:627947. doi: 10.3389/fonc.2021.627947 (PMC7968370; doi:10.3389/fonc.2021.627947)
Supplement: Supplementary file 1 [file DataSheet_1.docx]

**Article title**

Potential of radiomics in the identification of stage T3 and T4a esophagogastric junction adenocarcinoma based on contrast-enhanced CT images

**Supplementary Fig. 1** Flowchart of the patient selection and exclusion. Data in parentheses are the numbers of patients.

**Supplementary Table 1** Original data and SMOTE amplification data distributions

| Variables | Original data | | |  | SMOTE amplification data | | |
| --- | --- | --- | --- | --- | --- | --- | --- |
|  | Training | Testing | SUM |  | Training | Testing | SUM |
| T3 | 31 | 13 | 44 |  | 93 | 13 | 106 |
| T4a | 109 | 47 | 156 |  | 109 | 47 | 156 |
| SUM | 140 | 60 | 200 |  | 202 | 60 | 262 |

**Supplementary Table 2** Univariate logistic regression analysis of traditional features between training and testing set.

| Features | Testing set | Training set | p |
| --- | --- | --- | --- |
| n | 59 | 141 |  |
| Age (mean (SD)) | 63.97 (7.77) | 63.33 (8.01) | 0.604 |
| Gender = 1 (%) | 48 (81.4) | 112 (79.4) | 0.907 |
| Serosa Smooth = 1 (%) | 6 (10.2) | 19 (13.5) | 0.682 |
| Serosa Cords = 1 (%) | 5 (8.5) | 12 (8.5) | 1.000 |
| Fat Clear = 1 (%) | 6 (10.2) | 17 (12.1) | 0.890 |
| Serous Blurred = 1 (%) | 5 (8.5) | 14 (9.9) | 0.956 |
| Serosa Nodular = 1 (%) | 48 (81.4) | 110 (78.0) | 0.735 |
| Fat Blurred = 1 (%) | 48 (81.4) | 110 (78.0) | 0.735 |
| Fat Disappearance = 1 (%) | 32 (54.2) | 73 (51.8) | 0.871 |
| Necrosis = 1 (%) | 27 (45.8) | 62 (44.0) | 0.939 |
| Thickness (median [IQR]) | 18.44 [13.55, 21.84] | 18.06 [14.13, 23.02] | 0.795 |
| Longest Diameter (median [IQR]) | 59.24 [44.94, 69.70] | 57.71 [45.95, 72.63] | 0.646 |

Serosa Smooth: smooth serous surface; Serosa Cords: a few short cords of the serous surface; Fat Clear: the fat space around the tumor was clear; Serous Blurred: blurred serosa or short stripes <1/3 the total lesion area; Serosa Nodular: irregular or nodular shape of the serous surface; Fat Blurred: dense burr or banded infiltration of the surrounding fat space; Fat Disappearance: disappearance of the fat space between the serosa and peripheral vessels; Necrosis: cyst degeneration or necrosis; Thickness: tumor thickness; Longest Diameter: the longest diameter of the tumor.

**Supplementary Table 3** the data of intra-group correlation coefficient

| **Feature** | **ICC (95%CI)** |
| --- | --- |
| original_firstorder_90Percentile | 0.99 (0.98–1) |
| original_shape_Flatness | 0.95 (0.88-0.98) |
| wavelet.HLL_glszm_SizeZoneNonUniformityNormalized | 0.75 (0.71-0.79) |
| original_shape_Sphericity | 0.95 (0.89-0.97) |
| original_glszm_ZoneEntropy | 0.81 (0.64-0.9) |
| wavelet.LHL_firstorder_Uniformity | 0.93 (0.85-0.96) |
| wavelet.LHL_glszm_LowGrayLevelZoneEmphasis | 0.8 (0.74-0.86) |
| wavelet.HHH_firstorder_Kurtosis | 0.97 (0.94-0.99) |
| wavelet.HHH_firstorder_Mean | 0.9 (0.81-0.95) |
| wavelet.LLH_firstorder_Median | 0.97 (0.93-0.98) |
| log.sigma.5.0.mm.3D_glszm_GrayLevelNonUniformity | 0.86 (0.73-0.93) |

**Supplementary table 4** Retained important features in the original data and SMOTE radiomics data and beta value (regression coefficient) of the features.

|  | **Num** | **Features** | **Beta value** |
| --- | --- | --- | --- |
| Original data | 5 | log.sigma.1.0.mm.3D_glszm_GrayLevelNonUniformity |  |
|  |  | log.sigma.1.0.mm.3D_glrlm_RunVariance |  |
|  |  | wavelet.LHH_first order_Median |  |
|  |  | wavelet.LHL_first order_Median |  |
|  |  | wavelet.LHL_glcm_Idn |  |
| SMOTE amplification data | 11 | original_firstorder_90Percentile | 0.4019 |
|  |  | original_shape_Flatness | 0.7212 |
|  |  | original_shape_Sphericity | -0.3750 |
|  |  | original_glszm_Zone Entropy | 0.7411 |
|  |  | wavelet.HLL_glszm_Size Zone NonUniformity Normalized | -0.8410 |
|  |  | wavelet.LHL_first order_Uniformity | 0.7909 |
|  |  | wavelet.LHL_glszm_Low Gray Level Zone Emphasis | -1.2444 |
|  |  | wavelet.HHH_first order_Kurtosis | 0.6876 |
|  |  | wavelet.HHH_first order_Mean | 0.5683 |
|  |  | wavelet.LLH_first order_Median | 0.6423 |
|  |  | log.sigma.5.0.mm.3D_glszm_Gray Level NonUniformity | 0.6427 |

**Supplementary Table 5** The equations of 11 features.

| Feature name | Equation |  |
| --- | --- | --- |
| original_firstorder_90Percentile | The 90^th^ percentile of X | X: a set of $N_{p}$ voxel is included in ROI |
| original_shape_Flatness | $\sqrt{\frac{\lambda_{least}}{\lambda_{major}}}$ | $\lambda_{major}$ and $\lambda_{least}$ are the lengths of the largest and smallest principal component axes |
| original_shape_Sphericity | $\frac{\sqrt[3]{36\pi V^{2}}}{A}$ | A: surface area of the mesh in mm^2^;  V: volume of the mesh in mm^3^ |
| original_glszm_Zone Entropy | $-\sum_{i=1}^{N_{g}} \sum_{j=1}^{N_{s}} p\left( i,j \right)\log_{2} \left( p\left( i,j \right)+\epsilon\right)$ | $N_{g}$: number of discreet intensity values in the image;  $N_{s}$: number of discreet zone sizes in the image;  ϵ: an arbitrarily small positive number |
| wavelet.HLL_glszm_Size Zone NonUniformity Normalized | $\frac{\sum_{i=1}^{N_{g}} \left( \sum_{j=1}^{N_{s}} \boldsymbol{P}\left( i,j \right) \right)^{2}}{N_{z}^{2}}$ | $\boldsymbol{P}\left( i,j \right)$: size zone matrix |
| wavelet.LHL_first order_Uniformity | $\sum_{i=1}^{N_{g}} p\left( i \right)^{2}$ |  |
| wavelet.LHL_glszm_Low Gray Level Zone Emphasis | $\frac{\sum_{i=1}^{N_{g}} \sum_{j=1}^{N_{s}} \frac{\boldsymbol{P}\left( i,j \right)}{i^{2}}}{N_{z}}$ | $N_{z}$: number of zones in the ROI |
| wavelet.HHH_first order_Kurtosis | $\frac{\mu_{4}}{\sigma^{4}}$ | $\mu_{4}$: 4^th^ central moment |
| wavelet.HHH_first order_Mean | $\frac{1}{N_{p}}\sum_{i=1}^{N_{p}} \mathbf{X}\left( i \right)$ |  |
| wavelet.LLH_first order_Median | median gray level intensity within the ROI |  |
| log.sigma.5.0.mm.3D_glszm_Gray Level NonUniformity | $\frac{\sum_{i=1}^{N_{g}} \left( \sum_{j=1}^{N_{s}} \boldsymbol{P}\left( i,j \right) \right)^{2}}{N_{z}}$ |  |

**Supplementary Fig.2** ROC curves of the four models


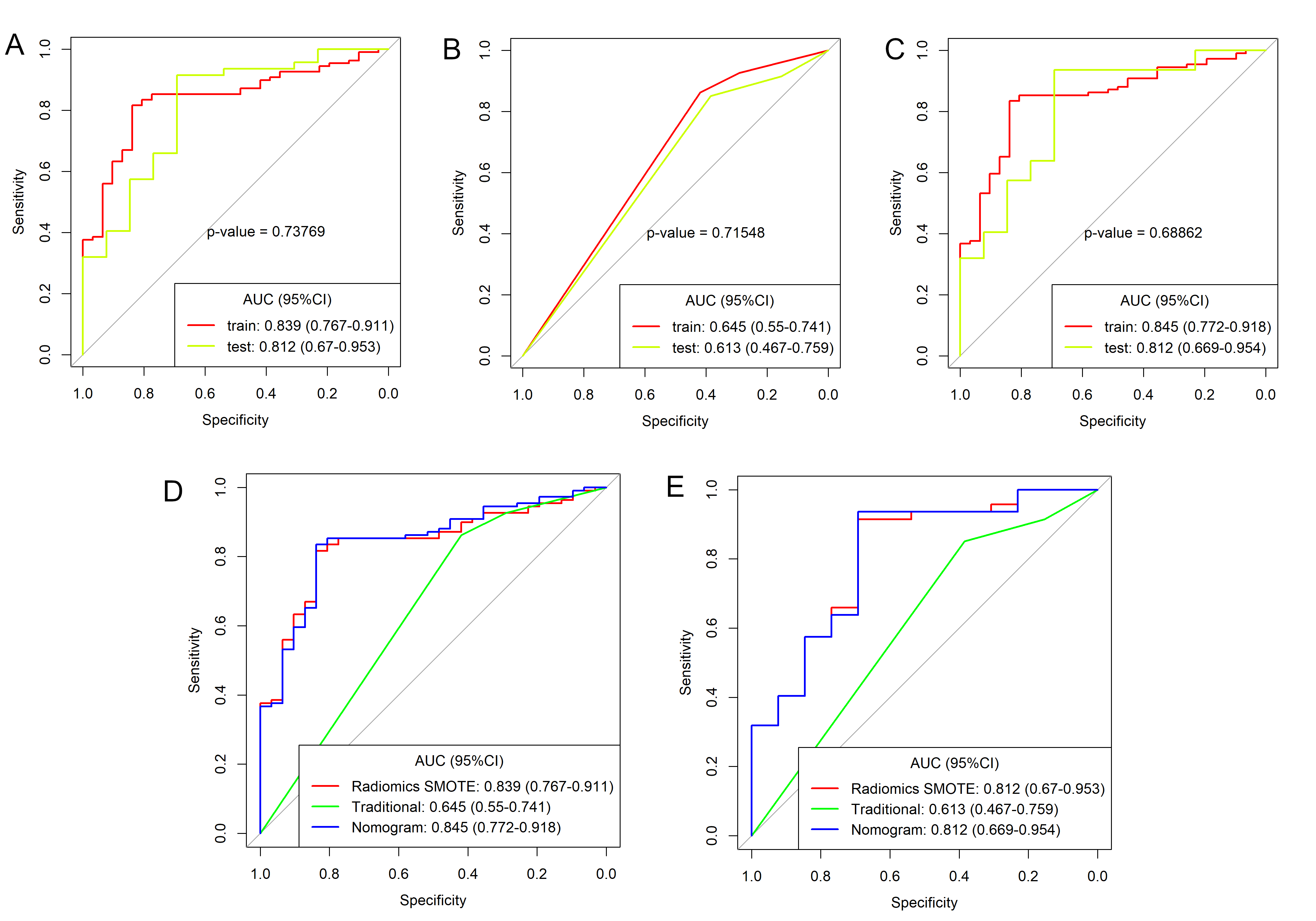


Receiver operating characteristic (ROC) curves comparing the identification power of the SMOTE radiomics model (A), the traditional model (B), and the combined nomogram (C) in the differentiation of T3 and T4a EGJ adenocarcinoma in the training set (red line) and the testing set (yellow line). ROC curves of the SMOTE radiomics model (red line), the traditional model (green line) and the combined nomogram (blue line) in the training set (D) and the testing set (E). The area under the curve (AUC) and 95% confidence interval (CI) for the models are shown in the lower right corner of the figure
